# Carboplatin May Be an Effective and Tolerable Treatment Option for Platinum-Sensitive Pancreatic Cancer Patients With Pre-existing Neuropathy

**DOI:** 10.7759/cureus.110034

**Published:** 2026-06-01

**Authors:** Angela Yoo, Jennifer Galvet, Sara Kim, Andy Choy, Daniel King

**Affiliations:** 1 Department of Oncology, Donald and Barbara Zucker School of Medicine at Hofstra/Northwell, Manhasset, USA; 2 Department of Pharmacy, Northwell Health Cancer Institute, New Hyde Park, USA; 3 Department of Radiology, Northwell Health, New Hyde Park, USA; 4 Department of Oncology, Northwell Health Cancer Institute, New Hyde Park, USA

**Keywords:** carboplatin, cisplatin, neurotoxicity, oxaliplatin, pancreatic adenocarcinoma, peripheral neuropathy, platinum

## Abstract

Platinum-based chemotherapy remains an important component of treatment for pancreatic adenocarcinoma; however, cumulative neurotoxicity from oxaliplatin and cisplatin often limits continued therapy. Carboplatin is a less neuropathic platinum analogue with activity in other malignancies, but it is not approved for pancreatic cancer. We describe a patient with pancreatic adenocarcinoma who achieved prolonged disease stability on carboplatin after discontinuation of oxaliplatin due to neuropathy.

A 57-year-old woman presented with pancreatic adenocarcinoma and was treated with neoadjuvant FOLFIRINOX (5-fluorouracil, leucovorin, oxaliplatin, irinotecan), chemoradiation, and pancreaticoduodenectomy, followed by adjuvant gemcitabine and capecitabine. Over time, she developed suspected locoregional recurrence and received multiple subsequent systemic therapies, including FOLFIRINOX rechallenge, 5-fluorouracil with liposomal irinotecan, and gemcitabine with nab-paclitaxel.

These regimens were limited by intolerance, disease progression, and worsening peripheral neuropathy. Given progression on non-platinum regimens and increasing neuropathy, but prior disease control with oxaliplatin, the patient was started on carboplatin. Initial imaging showed improvement in ascites and stabilization of retroperitoneal disease. She experienced radiographically stable disease for 13 cycles over approximately 10 months without worsening neuropathy before eventual disease progression.

This case suggests that carboplatin may be a reasonable option for select patients with pancreatic adenocarcinoma who appear to benefit from platinum therapy but cannot tolerate oxaliplatin or cisplatin due to neuropathy. While clinical data are limited, prior studies have demonstrated modest efficacy and a favorable toxicity profile with carboplatin-based regimens. Carboplatin may warrant consideration as an alternative platinum agent or as a switch-maintenance strategy in patients with platinum sensitivity and cumulative neuropathy.

## Introduction

Pancreatic adenocarcinoma is one of the most lethal solid malignancies, and systemic chemotherapy remains the foundation of treatment. Platinum-containing therapies, including FOLFIRINOX (5-fluorouracil (5-FU), leucovorin, oxaliplatin, irinotecan) [1], NALIRIFOX (liposomal irinotecan (nal-IRI), 5-FU/leucovorin, oxaliplatin) [2], and gemcitabine/cisplatin [3], are established therapeutic options that have demonstrated improvements in disease control and survival outcomes in appropriately selected patients. However, these regimens contain oxaliplatin or cisplatin, both of which are associated with cumulative neurotoxicity that may manifest as progressive peripheral neuropathy. Peripheral neuropathy can significantly impact quality of life and functional status, particularly with prolonged treatment duration, often necessitating dose reduction or early discontinuation of therapy. 

In patients who develop treatment-limiting neuropathy or intolerance to oxaliplatin-containing therapy, changes in treatment often involve transition to non-platinum regimens such as 5-FU and nal-IRI. As maintenance and switch-maintenance strategies have become increasingly important in the management of metastatic pancreatic cancer, there remains interest in identifying alternative platinum agents with improved tolerability profiles for patients who continue to derive benefit from platinum-based therapy. Carboplatin is an alternative platinum analog with activity similar to other platinum agents but with a more favorable neurotoxicity profile; however, it is not approved for pancreatic adenocarcinoma. This report describes a patient with pancreatic adenocarcinoma who achieved prolonged disease stability on carboplatin without worsening neuropathy after the discontinuation of oxaliplatin due to cumulative neuropathy.

## Case presentation

A 57-year-old woman presented in January 2020 with a 15-pound weight loss, obstructive jaundice, and a 1.8 cm mass in the uncinate process of the pancreas. She received nine biweekly cycles of neoadjuvant FOLFIRINOX followed by preoperative radiation and pancreaticoduodenectomy. In the adjuvant setting, she underwent four cycles of gemcitabine with capecitabine. Due to concern for local recurrence based on imaging findings of soft tissue haziness adjacent to the superior mesenteric artery, she underwent repeat radiation therapy with 36 Gy in three fractions. Eight months later, CA 19-9 levels began rising, and imaging demonstrated increasing retroperitoneal lymphadenopathy that was inaccessible to biopsy. Given concern for locoregional recurrence, FOLFIRINOX was reinitiated; however, treatment was discontinued after two cycles because of an oxaliplatin infusion-related reaction and neuropathy. She was subsequently treated with 5-FU/nal-IRI. After three cycles, she developed persistent diarrhea requiring electrolyte repletion along with radiographic progression, including worsening perivascular soft tissue involvement within the retroperitoneum and porta hepatis, as well as the development of large-volume ascites. She was then transitioned to gemcitabine/nab-paclitaxel (GnP), which she received for seven months before developing worsening neuropathy and progression of disease with increasing infiltration at the root of the mesentery. Neuropathy primarily involved the fingers and toes and progressed to grade 3 severity, significantly limiting the patient's activities of daily living and requiring treatment with gabapentin. Symptoms included impaired sensation and decreased dexterity, which interfered with the patient's fine motor function and contributed to minor injury during the course of treatment. Given the progression of disease on 5-FU/nal-IRI and GnP, along with worsening neuropathy, but prior disease control on oxaliplatin-containing therapy, it was hypothesized that the tumor retained sensitivity to platinum-based therapy.

Carboplatin was initiated with a target area under the curve (AUC) of 4 every 3 weeks under an inpatient desensitization protocol because of the patient's prior oxaliplatin infusion-related reaction. Follow-up imaging demonstrated decreased ascites, improvement in colonic wall thickening, and stable disease involving the pancreatic mass. The patient maintained radiographically stable disease for 13 treatment cycles over the course of 10 months before eventual progression (Figure 1). Although CA 19-9 levels gradually increased throughout treatment, the patient continued to demonstrate sustained radiographic stability and clinical benefit while receiving carboplatin. CA 19-9 increased from 15 U/mL prior to treatment initiation to 31 U/mL by cycle three, followed by a more gradual rise to 158 U/mL by cycle nine. A more pronounced elevation to 325 U/mL, following cycle 13, ultimately corresponded with disease progression. Trends in CA 19-9 levels throughout treatment are summarized in Figure 2.

**Figure 1:**
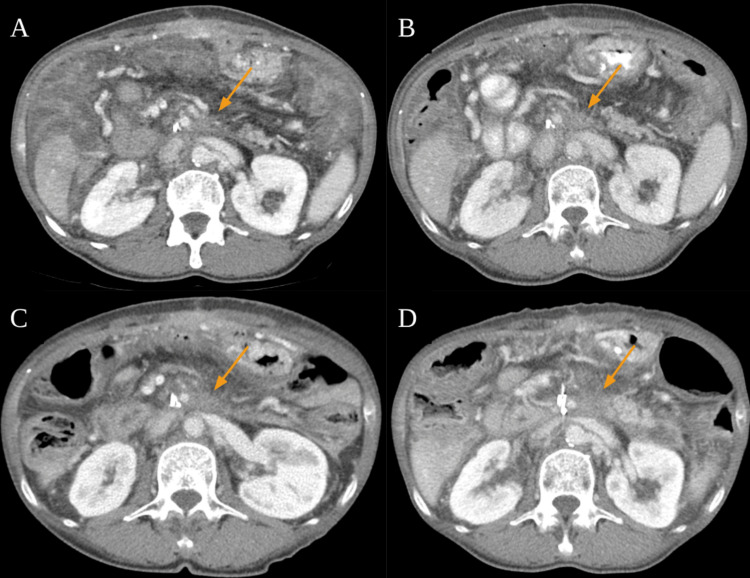
Serial axial CT images demonstrating radiographic stability of ill-defined mesenteric soft tissue following the initiation of carboplatin therapy (A) Baseline CT imaging obtained at treatment initiation (Apr 2023, Cycle 1); (B) Early-treatment CT imaging obtained after Cycle 3 (May 2023); (C) Late-treatment CT imaging obtained after Cycle 9 (Oct 2023); (D) Post-treatment CT imaging obtained after Cycle 13 (Jan 2024)

**Figure 2:**
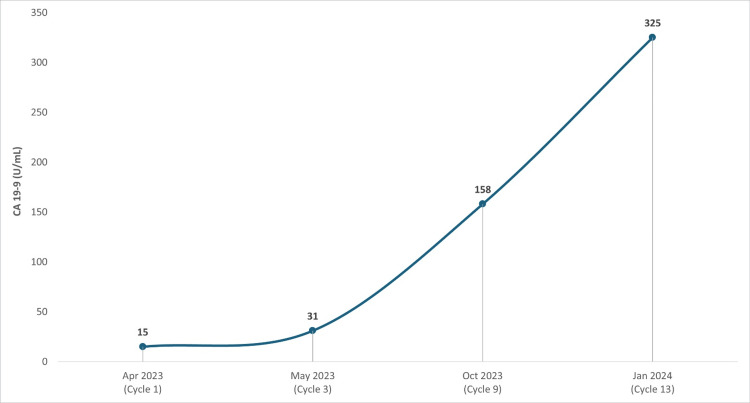
Changes in CA 19-9 levels across treatment cycles during carboplatin therapy

## Discussion

Pharmacology

Platinum Neurotoxicity

The three platinum compounds available in the United States are cisplatin, carboplatin, and oxaliplatin. While these platinum compounds share structural similarities, their therapeutic uses, pharmacokinetics, and toxicity profiles vary (Table 1). Platinum compounds cause DNA damage by forming intrastrand DNA adducts and interstrand DNA crosslinks, resulting in impaired DNA synthesis, transcription, and cellular function, ultimately triggering apoptosis [4-6]. Chronic peripheral sensory neuropathy associated with platinum compounds results from injury to sensory neurons within the dorsal root ganglia, and the degree of neurotoxicity has been shown to correlate directly with the amount of DNA cross-links found in dorsal root ganglion neurons in mouse models injected with cisplatin [5,7]. Symptoms are predominantly characterized by numbness, tingling, and paresthesia in the extremities and may progress to loss of deep-tendon reflexes and leg weakness with gait disturbance [8]. Acute neurotoxicity is largely limited to oxaliplatin and is thought to result from heightened neuronal excitability and changes in axonal refractoriness through interactions with voltage-gated sodium channels, resulting in altered sodium channel inactivation kinetics [7,9].

**Table 1:** Comparison of pharmacokinetics and toxicities of platinum agents Carboplatin AUC-based dosing is calculated using the Calvert formula: Total dose (mg) = Target AUC x (GFR + 25) GFR is typically capped at 125 mL/minute. Pharmacokinetic and toxicity data adapted from previously published literature [14,16].

Pharmacologic Characteristic	Cisplatin	Carboplatin	Oxaliplatin
Pharmacokinetics			
Protein binding	>90%	0%	>90%
Renal elimination	Up to 17% as cisplatin; 50% as platinum-containing metabolite	75%	54%
Half-life	Cisplatin: 20-30 minutes; Platinum: ≥ 5 days	Carboplatin: Up to 6 hours; Platinum: ≥ 5 days	392 hours (16 days)
Dose range	20-100 mg/m2	300-900 mg*	60-130 mg/m2
Dose-limiting toxicities	Nephrotoxicity; Neurotoxicity (neuropathy and ototoxicity)	Myelosuppression	Peripheral neuropathy
Major toxicities: Relative risk			
Myelosuppression	+	+++	+
Nephrotoxicity	+++ (dose-dependent)	+	+
Peripheral neuropathy	+++	+	+++
Ototoxicity	+++	+	+
Emetogenic potential	+++	++ to +++ (dose-dependent)	++

Common toxicities of platinum compounds include nausea and vomiting, myelosuppression, neurotoxicity, and nephrotoxicity, with varying frequencies among agents. As summarized in Table 1, cisplatin and oxaliplatin are associated with substantially higher rates of peripheral neuropathy compared to carboplatin, whereas carboplatin is more commonly associated with myelosuppression. Cisplatin additionally carries greater risks of nephrotoxicity and ototoxicity. Oxaliplatin is also uniquely associated with acute cold-induced neuropathic symptoms as discussed below. These differences are important when selecting platinum-based therapy, especially in patients with pre-existing or progressive neuropathy who may not tolerate continued exposure to oxaliplatin or cisplatin.

Cisplatin

Cisplatin is a first-generation platinum analog that has demonstrated activity against various types of malignancies [8]. Its dose-limiting toxicities include nephrotoxicity and neurotoxicity, including cumulative peripheral neuropathy and ototoxicity. Peripheral neuropathy generally occurs at cumulative doses exceeding 300 mg/m2 and develops in nearly all patients at cumulative doses of 600 mg/m2 [2,8]. Cisplatin-induced neuropathy can present weeks after the last dose and can worsen over one to six months [10,11].

Carboplatin

Carboplatin, a second-generation platinum analog, was developed to reduce the dose-limiting toxicities of cisplatin [8]. Among the platinum compounds, carboplatin carries the lowest risk of peripheral neuropathy (4-6%), and clinically significant neuropathy is uncommon at conventional doses [12]. Reported cases of severe neuropathy have primarily occurred in patients receiving high-dose carboplatin (cumulative doses up to 2,750 mg/m2) in combination with etoposide and ifosfamide in the hematopoietic stem cell transplant setting who had baseline neuropathy from prior cisplatin exposure (cumulative doses up to 450-800 mg/m2) [13]. For an average patient receiving carboplatin AUC 4 every three weeks, neuropathy is considered a delayed, cumulative toxicity that would be expected only after prolonged exposure. Reaching cumulative levels associated with severe neuropathy would require approximately 12-13 cycles, corresponding to approximately 8 to 9 months of therapy. By comparison, the median progression-free survival associated with these regimens is approximately six months, suggesting that many patients would discontinue carboplatin before neuropathy becomes clinically significant. These rare occurrences of severe neuropathy following high-dose carboplatin may instead reflect delayed-onset cisplatin-induced neuropathy, which typically manifests weeks after cisplatin discontinuation and progresses over several months without a direct causal relationship to carboplatin [10,11].

Oxaliplatin

Oxaliplatin is a third-generation platinum analog that was developed to circumvent resistance against cisplatin and carboplatin [8]. The presence of the bidentate oxalate ligand reduces reactivity and may contribute to a more favorable non-neurologic toxicity profile [7]. The dose-limiting toxicity of oxaliplatin is peripheral neuropathy, which can occur as two distinct forms. Acute peripheral sensory and/or motor toxicity typically develops during or within 72 hours of administration and usually resolves spontaneously within hours. Symptoms may include cramps, muscle spasms, jaw spasm, and cold-induced transient paresthesias involving the mouth, larynx/pharynx, and extremities [14,15]. Chronic peripheral sensory neuropathy typically occurs with cumulative doses of 700 mg/m2 or greater and may not be completely reversible, although severity improves over time. Chronic neuropathy typically presents with persistent dysesthesias, paresthesias of the extremities, and impaired fine sensory-motor coordination [14,15].

Clinical relevance in pancreatic cancer

Carboplatin exhibits a more favorable toxicity profile than cisplatin and has therefore replaced cisplatin in the treatment of several malignancies in which similar efficacy has been demonstrated, including lung and ovarian cancers [7,16-18]. However, carboplatin has demonstrated inferior clinical efficacy compared with cisplatin in the treatment of testicular cancer, head and neck cancer, bladder cancer, and esophageal cancer; therefore, cisplatin remains the preferred platinum agent for these malignancies [8,15]. In pancreatic cancer, both oxaliplatin and cisplatin have shown efficacy. Although carboplatin is not approved for pancreatic adenocarcinoma and is not widely used in this setting, limited clinical data suggest potential activity, with response rates of approximately 20% reported for gemcitabine plus carboplatin compared with approximately 5% for gemcitabine monotherapy [19,20].

A phase II clinical trial published in 2005 evaluated the efficacy and tolerability of gemcitabine plus carboplatin in patients with advanced pancreatic cancer [19]. Among the 35 evaluable patients, 8 (17%) achieved partial response, 15 (32%) patients had stable disease, and 12 (25%) experienced progressive disease [19]. Neuropathy was not reported as a treatment-related toxicity, and grade 3-4 adverse events were limited primarily to hematologic toxicities [19]. The regimen was overall well-tolerated and associated with improvements in Karnofsky performance status and weight. A more recent retrospective study published in 2023 evaluated the efficacy and tolerability of leucovorin and 5-fluorouracil (LV5FU2) and carboplatin in patients with advanced pancreatic ductal adenocarcinoma following progression on standard therapies [5]. This regimen was administered for an average of three cycles and used mainly in the third-line (59.3%) and fourth-line (23.1%) settings. The clinical benefit rate, defined as the proportion of patients achieving objective response or disease stabilization, was 25.2%. Importantly, worsening of pre-existing neurotoxicity occurred infrequently (13.2%) [5]. These findings suggest carboplatin may retain antitumor activity while offering a more favorable neurotoxicity profile. 

There are inherent limitations to evaluating neuropathy, as assessment is largely subjective and based on patient-reported symptoms rather than objective quantitative measures. However, because neuropathy is a clinically significant toxicity that frequently limits treatment tolerability, consideration of alternative treatments remains important. As maintenance strategies have become increasingly important in the management of metastatic pancreatic cancer, carboplatin may represent a potential switch-maintenance therapy for patients who continue to benefit from platinum-based therapy but develop cumulative neuropathy. It may also serve as an alternative platinum agent in patients with pre-existing neuropathy.

## Conclusions

This case suggests that carboplatin may represent a reasonable treatment option for select patients with platinum-sensitive pancreatic adenocarcinoma who are unable to tolerate oxaliplatin or cisplatin due to cumulative neuropathy. In this patient, carboplatin was associated with prolonged radiographic disease stability without worsening neuropathy despite extensive prior therapy and pre-existing neurotoxicity. Although clinical data remain limited, prior studies have demonstrated modest efficacy and a comparatively favorable neurotoxicity profile with carboplatin-based regimens. Carboplatin may also represent a potential switch-maintenance strategy in patients who continue to derive benefit from platinum-based therapy but develop cumulative treatment-limiting neuropathy.

Although neuropathy assessment remains largely subjective and patient-reported, treatment-related neurotoxicity can significantly impact quality of life and limit continued therapy. Carboplatin may provide an alternative platinum option for carefully selected patients requiring ongoing platinum-based treatment in the setting of pre-existing or progressive neuropathy. Prospective studies are warranted to further evaluate carboplatin-based treatment options in pancreatic adenocarcinoma, particularly among patients with biomarkers or clinical features suggestive of platinum-sensitive disease.
